# Effects of Multi-Ingredient Pre-Workout Supplement and Caffeine on Bench Press Performance: A Single-Blind Cross-Over Study

**DOI:** 10.3390/nu14091750

**Published:** 2022-04-22

**Authors:** Marek Kruszewski, Maciej Merchelski, Artur Kruszewski, Rafał Tabęcki, Maksim Olegovich Aksenov, Łukasz Pągowski

**Affiliations:** 1 Department of Physical Education, Józef Piłsudski Univesity of Physical Education in Warsaw, 00-809 Warszawa, Poland; artur.kruszewski@awf.edu.pl (A.K.); rafal.tabecki@awf.edu.pl (R.T.); luk.pagowski@gmail.com (Ł.P.); 2 Department of Physical Education, Plekhanov Russian University of Economics, 36 Stremyanniy, 117997 Moscow, Russia; 3 Department of Physical Education, Banzarov Buryat State University Ulan Ude, 24a Smolina, 670000 Buryatia, Russia

**Keywords:** multi-ingredient pre-workout supplement, ergogenic aids, sports nutrition, pre-workout supplementation, caffeine, resistance exercise, strength endurance

## Abstract

The problem addressed in this study is the appropriateness of using different pre-training supplementation strategies and their ability to improve training performance and psychological measures. The aim of the study is the evaluation of the effectiveness of a multi-ingredient pre-workout supplement (MIPS) containing beta-alanine, L-citrulline malate, arginine alpha-ketoglutarate, L-taurine, L-tyrosine and caffeine compared to an exact dosage of anhydrous caffeine in bench press strength endurance, feeling scale (FS), felt arousal scale (FAS) and session rating of perceived exertion (sRPE). A group of fifteen resistance-trained males, weighing 83.92 ± 8.95 kg and having an average of 5.6 ± 3.38 years of training experience, tested their bench press 10 repetition maximum (79.01 ± 12.13). In a cross-over manner, they participated in two sessions where they were blinded to the order of supplementation they were given: either a MIPS including caffeine or caffeine alone. They completed the bench press strength endurance test with pre- and post-training psychological assessments containing FS, FAS and sRPE. Bench press repetition volume was greater after anhydrous caffeine than MIPS supplementation with no difference in psychological measures. These results indicate that MIPS supplementation is less ergogenic and cost effective than caffeine alone.

## 1. Introduction

In 2019, the world’s dietary supplements market was estimated to have a value of over 123 billion USD, and experts predict an upward trend into the future. It is forecast that the global market’s value will almost double and reach 230 billion USD by 2027 [1]. Dietary supplements are also commonly used in Poland, where their market was valued at around 1 billion USD in 2017 [2]. A significant segment of the supplement market is taken by sports supplements. As well as single-ingredient supplements, many are sold as combinations.

A popular example of such is multi-ingredient pre-workout supplements (MIPSs). Their effects may be acute (ergogenic and neurogenic) or long term (improved training adaptations) [3]. The most popular amongst them are [4]: beta-alanine; caffeine; citrulline; tyrosine; taurine; creatine; niacin; arginine; vitamin B12; betaine; choline bitartrate; theanine; BCAAs; carnitine; yohimbe; and beetroot extract. 

No ergogenic effect of MIPSs was observed in four studies. In two of them, upper-body multi-joint exercises were employed—pushups [5]—and a standardized YMCA (Young Men’s Christian Association) bench press test and curl-up test [6]. In the other two studies, single-joint exercises were used—alternating isokinetic concentric contractions of the elbow flexors and extensors [7]—and leg extensions [8]. In the rest of the literature, MIPSs’ ergogenic effect was observed for upper- and/or lower-body exercise [9,10,11,12,13,14,15,16].

Regarding long-term studies, in three cases, supplementation had positive effects on muscle hypertrophy and strength gains after a 28-day training intervention [15,17,18]. In one paper, there was a positive effect only on muscle hypertrophy after 8 weeks of resistance training [19]. In one study, no positive effect of supplementation was found [20], even though the supplement contained caffeine and was previously confirmed [17,18]. In the last study, ergogenic effect was not found; however, the MIPS did not contain caffeine [20].

In three papers, subjective energy levels were assessed. In two of them, MIPS supplementation caused participants to feel more energetic [11,14]. In the third case, no positive effect on perceived energy was noted, even though positive effect on muscle power was observed [10].

The most widely researched of MIPS ingredients is caffeine, and its positive effects on performance were confirmed by an umbrella review [21]. The positive effects of caffeine on strength training performance were also confirmed in woman, whose participation in earlier research was scarce [22]. Recently, the positive response to caffeine was linked to the CYP1A2 and ADORA2A genes. Even though they may influence the effect size of caffeine, its positive effects were confirmed for all their variations [23,24].

Contrary to the acute effect of caffeine, creatine must be supplemented for a period of time before its effects can be observed [25,26,27]. Its positive effects on strength training performance are widely reported in literature [28,29,30,31]. What is more, creatine supplementation can lead to positive effects on muscle hypertrophy by increasing muscle protein synthesis through stimulating mRNA translation [28,32] and/or elevation of muscle cell hydration [33,34].

Beta-alanine, also often included in MIPSs, is used in supplements in order to increase muscle carnosine levels. Carnosine is reported to serve in muscle tissue as a physiological buffer. Similar to creatine, a supplementation period usually lasting about 28 days must be implemented before its effects can be observed. Doses recommended in the research span from 2 up to 6 g [35]. Beta-alanine supplementation seems to be ergogenic in activities lasting from 30 s to 10 min. However, the effectiveness of its supplementation in a resistance exercise context is unsure. Similar to creatine, there is a possible direct effect of beta-alanine supplementation on muscle hypertrophy caused by its osmotic properties [34,36,37,38,39].

The supposed influence of tyrosine on adrenaline and dopamine levels has not been satisfactorily proven so far nor has its effect on exercise performance been confirmed [40,41]. However, research without an exercise component suggested positive effects on cognitive performance in stressed conditions with a bigger tyrosine dosage [42].

Taurine supplementation may increase post-exercise lipid oxidation and was shown in some research to improve post-exercise recovery [43,44]. It also showed ergogenic properties in low- and moderate- but not high-intensity exercise [45,46,47].

MIPSs also often contain various nitric oxide boosters which are claimed to positively affect training performance by improving blood flow. Among nitric oxide boosters, we can count arginine, as well as its precursor, citrulline, and beetroot juice and its ingredient betaine [48,49]. The ergogenic effect of arginine was observed for aerobic as well as anaerobic exercise [50]. Citrulline intake is known to cause more significant blood arginine concentration than arginine supplementation. Besides its effects on muscle endurance, citrulline may also positively affect muscle strength [50,51,52]. Beetroot juice supplementation showed positive effects on endurance, as well as intermittent, high-intensity exercise performance [53,54]. Research also demonstrated the ergogenic effects of betaine supplementation on aerobic [55] but not resistance exercise [56].

The current state of knowledge highlights the importance of academic research on sports supplements. We deem it prudent to investigate effects of various co-supplementation strategies used in both professional and recreational sports. The fulfillment of such a mission and apparent deficiencies in MIPS literature led to the formulation of the main, as well as the secondary, aim. The main aim of this study is to compare the effectiveness of MIPS and caffeine anhydrous supplementation in bench press strength endurance, feeling scale (FS), felt arousal scale (FAS) and session rating of perceived exertion (sRPE). This study’s secondary aim is to compare the financial efficiency of the described pre-workout supplementation strategies.

## 2. Materials and Methods

### 2.1. Participants

Fifteen participants with a mean body mass of 83.92 ± 8.95 kg and 5.6 ± 3.38 years of resistance training experience participated in the experiment. Participants had to fulfil the following criteria: be male, between 16 and 40 years old; have at least 1 year of bench press training experience; be able to complete 10 bench press repetitions with at least 75% body mass; be of good health, free of injuries; have no allergies, intolerances or history of side effects to any of the MIPS’s ingredients; be able to maintain positive or neutral energy balance for the duration of the study and at least 1 week prior. Participants were instructed to maintain their regular lifestyle and refrain from any additional physical activities. No participant was involved in any hard physical work nor was working nightshifts. Participants were required to abstain from regenerative treatments (sauna, massage, hydrotherapy, etc.) and supplementing with pharmacological or nutritional agents (performance-enhancing drugs, nutrients, vitamins, etc.) and to maintain their nutritional habits for the duration of the study. All appropriate consents were signed. The measurements were conducted in the Department of Individual Sports in Combat Sports and Weightlifting Section, Józef Piłsudski University of Physical Education in Warsaw, Poland, in the Laboratory of Muscle Strength. The team with the necessary qualifications and experience in research performed the measurements on the entire study group. Participants were informed of the nature of the investigation with a clear statement of the objective of the research and possible risk. They could withdraw at any time during the study. Written informed consent was obtained prior to study commencement. The experimental protocol was approved by the Commission for Ethics of Scientific Research at Józef Piłsudski University of Physical Education in Warsaw (protocol number SKE 01–06/2021), Poland, and conformed to the principles presented in the Declaration of Helsinki.

### 2.2. Protocol

Data collection was performed during 3 visits. Participants were acquainted with the study participation criteria and testing protocol on the first visit. Participants’ bench press 10 repetition maximum was measured. No substance was supplemented on the first visit. After at least 48 h, in a cross-over manner, participants took part in 2 training sessions where they were given either a MIPS or caffeine. Thirty minutes after supplementation, participants performed 10 min of standardized, dynamic warm up, followed by a specific bench press warm up. Participants performed 5 bench press sets to failure with the load established at the first visit, allowing for completion of 10 repetitions and a 180 s rest period. Training sessions were performed at the same time of day with an interval of at least 2 h from consumption of a standardized meal. Training sessions were separated by at least 72 h. After dynamic warm up and 2 min after the last bench press set, a verbal psychological questionnaire was conducted.

### 2.3. Training Load Determination

In order to establish the weight of the barbell used in ergogenic testing, the maximal load with which participants were able to complete 10 bench press repetitions was measured. Participants performed 10 min of standardized, dynamic warm up followed by bench-press-specific warm up. Bench press sets of 10 were performed, and, after every set, participants reported RIR (repetitions in reserve) at the end of each series—the number of repetitions that the exerciser would be able to perform while continuing the set [57]. Based on the reported RIR, the load was increased by 2.5 kg or its multitude. Sets were performed with 180 s rest periods until participants were unable to complete 10 repetitions. Barbell mass used in the last, fully completed set of 10 was considered as the result of the test. Bench pressing was performed accordingly to IPF (International Powerlifting Federation) rules. Sets were terminated when participants were unable to finish a repetition or made a technical error on 2 consecutive repetitions. The technical error was understood accordingly to the IPF rulebook [58].

### 2.4. Method of Training Load Assessment

Training loads performed during ergogenic testing were measured in effective work value (Lu), a product of barbell mass (kg) and displacement (cm) measured in kGm and converted into SI system unit KJ. To compare results obtained by subjects with different body weights, values were scaled to 1 kg of body mass. Height of elevation was related to participants’ height, in line with the methodology of N. N. Saksonow modified by M. Kruszewski [59]. Volume load was counted as effective work (product of barbell weight and displacement) achieved during bench press where barbell displacement was set to 24.7% of body height [60].

### 2.5. Psychological Measures—Feeling Scale, Felt Arousal Scale and Session Rate of Perceived Exertion

State of mind was measured with an 11-point bipolar good/bad feeling scale (FS) ranging from +5 to −5, where +5 described a very good and −5 a very bad state of mind [61]. Arousal was measured with a 6-point felt arousal scale (FAS), where 1 described low and 6 high arousal [62]. Exhaustion was measured with an 11-point session rate of perceived exertion scale (sRPE) created by Foster et al. [63] and validated by Haddad et al. [64], where 0 was rest and 10 was maximal effort.

### 2.6. Dosage and Supplementation Method

Supplements were served in liquid form. Participants were blinded to the supplementation condition. The caffeine dose was individualized to 6 mg/kg of body weight: the dose most commonly used in the literature [65]. MIPS dose was equalized to 6 mg/kg of body weight of caffeine. The supplemented MIPS was a popular, commercially available pre-workout supplement with relatively high caffeine content—300 mg per serving (most products contain 50% less caffeine per serving). The supplement’s suggested serving (12.5 g) and mean doses of specific ingredients used in the study are presented in Table 1.

### 2.7. Market Query

E-commerce service “Allegro.pl” was queried for MIPS and caffeine supplements. The following product details were gathered: suggested portion size, caffeine content per serving and serving price. Query was carried out in the subcategory “pre workout supplements”. In order to avoid disturbances, results were filtered. For the MIPS query, in order to exclude product samples and bundles, product mass was narrowed to 100–1000 g. Only MIPSs in the most commonly used powder form were taken into account. To eliminate singular offers of imported and new-to-market products, results were filtered to customer-reviewed products. All supplements that contained ingredients listed on the WADA (World Anti-Doping Agency) prohibited list were excluded [66]. Caffeine supplements were filtered by form. Supplements in pill form were gathered together as they represented the same method of supplementation. A separate query was carried out for caffeine in powder form. Only supplements containing nothing but caffeine anhydrous were included.

### 2.8. Statistical Analysis

Dependent sample *t*-test was employed to compare MIPS and caffeine supplementation for: serving price; bench press repetition volume in first, second, third, fourth and fifth set; total bench press repetition volume; pre- and post-bench-press FS, FAS and sRPE. To measure between MIPS and caffeine conditions effect sizes, Cohen’s d was calculated for performance, as well as for psychological measures. Effect sizes over 0.2 were classified as small, over 0.5 as medium and over 0.8 as large [67].

## 3. Results

### 3.1. Effects of Supplementation

There was no significant effect of supplementation order on volume performed (*p* = 0.89, d = 0.02). A comparison of supplementation conditions, bench press volume and psychological measures is presented in Table 2. Independent from the supplementing conditions, there was a clear downtrend for set repetition volume. Significant difference was found for the third set (*p* = 0.02) and total repetition volume (*p* = 0.04) with a small effect size (d > 0.2).

There was no difference in the effect of caffeine and MIPS ingestion on pre- and post-training psychological measures (*p* > 0.05) (Table 3).

### 3.2. Price Comparison

Market query resulted in 101 different MIPSs being found (Table 4). Eight products were excluded. Five exclusions were due to a lack of information on ingredient dosages, and three were caused by inaccuracy in supplement facts. The remaining MIPSs were divided by distribution, where 57 were locally distributed and 36 were imported. The query of caffeine supplements resulted in 21 products in pill form and two products in powder form (Table 4).

The mean suggested portion price was significantly higher for imported than locally distributed MIPSs (*p* = 0.0024). Therefore, we decided to exclude imported MIPSs from the consequent analysis. Caffeine price per serving was normalized and compared to MIPS price per serving. Significant differences in serving prices were found between MIPSs and caffeine both in pill form (*p* < 0.01) and powder form (*p* < 0.01). The results show that, for the price of one portion of MIPS, it is possible to buy 10 portions of caffeine in pill form or 50 portions of caffeine in powder form.

## 4. Discussion

The results of our research indicate that the potential ergogenic benefits of MIPS supplementation may be negated through antagonist relations between its specific ingredients. Even though specific, commonly included substances in MIPSs have been described by a relatively vast body of research, our knowledge of their interactions is very scarce. The possibility of disturbances of the natural physiological process by the action of supplementing is sometimes indicated [68]. The issue of potential synergistic and antagonist effects of different substances were studied as far back as the 1990s, when the negation of creatine’s ergogenic benefits by caffeine was observed [69]. This, and subsequent research investigating effects of creatine and caffeine co-supplementation, is the showcase example of the possibility of negative interactions between various supplements. 

Caffeine’s main mechanism of action is based on the inhibition of adenosine receptors and stimulation of adrenaline secretion. This results in increased arousal, pain tolerance and fatigue tolerance. The effects of caffeine may go beyond CNS and apply directly to respiratory, circulatory and muscular systems [70,71]. The opposite actions of caffeine and creatine on muscle relaxation time is a known mechanism [72]. However, in most studies where the effects of creatine were negated by caffeine co-supplementation, there were reports of gastric issues in some participants [68]. Dosages used in creatine loading protocols described in the literature amounted to as much as 30 g a day. After the loading period, dosages of 2–5 g were sufficient to maintain elevated muscle creatine levels [25,26].

Creatine dosages used in MIPSs, usually between 3–6 g, can lead to full muscle creatine saturation in 21–28 days. Creatine is phosphorylated to creatine phosphate, a substrate in ATP desensitization. Supplementation of creatine was shown to be ergogenic mainly in efforts lasting up to 30 s and, to a lesser degree, 150 s [28,29,30]. However, in high doses it may cause gastric issues. This, in turn, may be a cause of diminished performance in subsequent testing. One paper investigated changes in muscular strength and body composition in four supplementation conditions: creatine, caffeine, creatine + caffeine and placebo. The only significant change in muscle strength and body composition after a 6-week training protocol occurred for knee extensors in the creatine supplementation group [73]. Although a low number of participants (caused by the COVID-19 pandemic) created uncertainty about the results of this study, it certainly raises some doubts.

Uncertainties surrounding the effects of co-supplementing even the two most widely used substances in strength training highlight the issue of possible interactions between other MIPS ingredients. Our knowledge of potential competitive absorption pathways or mutually exclusive effects of specific MIPS ingredients is limited. The existence of other such interactions would explain the weak ergogenic and arousing effects of the MIPS observed in our study. From this point of view, supplementation with MIPSs seems to be a risky bargain. We are taking on a risk of possible negative interactions between its ingredients, as well as financial loss from paying for substances that diminish the overall effects of supplementation.

When reviewing research regarding MIPSs, there are couple of important issues to be acknowledged. Researchers may employ various protocols designed to highlight specific aspects of MIPS effects but not always include reliable muscle endurance testing, which may render it impossible to make any comparisons in this regard [74,75,76,77]. Another substantial issue with MIPS research is a high variability of its ingredient profiles. It is hard to find instances of the same product being investigated repeatedly, while both ingredients and portion sizes are highly varied between different MIPSs. Unfortunately, such information may not even be known, as many MIPSs consist of proprietary blends [78]. The use of such labelling takes the responsibility to disclose exact concentrations of specific ingredients off the producers. Research of such supplements would be of no use in analyzing correlations between MIPSs’ effects and their ingredient profiles. Although evidence of MIPS effects on acute training performance and arousal, as well as, to a lesser extent, long-term muscle hypertrophy, is mostly positive, low levels of consistency and reliability make it too early for a firm recommendation [51].

Inconsistencies in the effects of MIPSs observed in previous research may be rooted in the different ingredient profiles of the investigated MIPSs. The supplement used in our study contained two substances (L-citrulline and L-taurine) that were not present in MIPSs in previous research [7]. We must consider the possibility of negative interactions between caffeine and one of these ingredients. Taurine is one of the most popular additives to caffeinated energy drinks, and their interactions were already investigated [79]. Although authors of this study suggested the possibility of taurine reducing caffeine’s effects on the cardiovascular system, such interactions were not confirmed by exercise trials. Previous research on MIPSs’ ergogenic effects may partially answer the question of the possible interaction of caffeine with L-citrulline and L-taurine. Amongst MIPSs investigated in previous research, two contained taurine and citrulline [11,16], three contained taurine but not citrulline [9,10,14] and two contained citrulline but not taurine [8,13] and, in four, neither was included [5,6,12,15]. Since, in the two papers where no ergogenic benefits were reported [5,6], none of the said substances was included in the investigated MIPSs, the probability of complete negation of caffeine’s positive benefits by one of them does not seem plausible.

Proponents of MIPS have different arguments in favor of their efficacy. MIPSs are claimed to be a convenient form of supplementation, with many substances collected in one well-formulated dose. They also praise their broad spectrum of effects—from acute ergogenic and arousing effects to long-term anabolic and thermic (supporting fat mass reduction) effects. In contrast to our position, they advocate for potential synergisms between specific ingredients of MIPSs [80].

However, the presence of substances requiring prolonged supplementation periods, such as creatine and beta-alanine, in MIPSs seems ambiguous. Laymen may be aware of the positive effects of these substances without knowledge of the required supplementation period. Companies may take advantage of this incomplete knowledge by including such ingredients in their products. It is expected that, in the free market economy, the positive effects of supplements are essential for sports supplement producers. Unfortunately, they usually settle for popular ingredient profiles or engage in an arms race with authorities by creating subsequent products containing ingredients banned by the WADA. Research groups monitoring the American supplement market, from which products are often imported or copied for the Polish market, state that producers do not discard such substances even after the intervention of the control body [81,82]. It creates need, or even a necessity, for cooperation with firms that maintain high standards of manufacturing and respect anti-doping rules. The particular importance of the issue gets accentuated when MIPSs are used in order to the aid the training effectiveness of competitive athletes subjected to anti-doping testing [83].

Since this issue was also relevant to our research, all substances used in our experiment were confirmed to comply with production, legal and ethical standards. When comparing the acute effects of MIPS and caffeine in volume performance, we had the certainty of reliability of supplemented substances and, therefore, credibility of results. Hence, any discrepancies between our results and those observed in other studies cannot be explained by any lack of so-called “purity” of substances used in our experiment. 

In the case of strength endurance tests results, causes of significant differences in effects may be found in the diversity of training loads, exercise performed and participants’ training experience. In our research, load amounted to 75% (1RM), sets were performed to muscular failure and inter-set rest periods were relatively long—180 s, as prescribed in the literature [60]. In comparison to other research, training intensity was 35% greater and inter-set rest periods were 120 s longer, which led to accentuation of the strength component over the endurance component [7,84]. A significant drop in volume performed between the first and subsequent sets suggests profound depletion of phosphocreatine stores, which can be useful in the translation of our results to other sports activities with similar levels of intensity [59]. In our research, significant differences in performed repetitions were observed in the third set. The lack of significant differences in earlier sets of the exercise may suggest null effects of supplementation in aspects of acute muscle strength and power. Effects of MIPS were rarely detected for muscle strength [11,12] and power [11,12,14]. However, even for caffeine, significant benefits were reported so scarcely that they were only confirmed by metanalyses [85]. In our experiment, no direct measurements of these characteristics were performed; however, the possibility of their increase seems doubtful in the context of our design.

The lack of significant differences observed during the first two sets may also be explained by relatively high muscle phosphocreatine concentrations present at that stage and during the final sets due to extreme exhaustion. It is essential to highlight the low probability of one set explaining differences observed in total volume performed. Cumulation of smaller differences in volume performed over the entire training protocol is more probable. Reliable investigation of effects in every single set would, however, have required a much larger sample size. Such an experiment could establish a correlation between supplementation effects and energy sources used at particular stages of the workout. For this purpose, comparison of velocity loss curves after supplementation of different supplements could prove advantageous [86].

Regarding the influence of training experience on ergogenic tests results, it was clearly prudent to expect trained participants in our research to achieve significantly greater training volumes than recreationally active participants [7,84]. Comparison of results is not, however, straightforward since it is confounded by differences in supplementation protocol—different MIPSs and dosages strategies of MIPS and caffeine. In our research, MIPS and caffeine were adjusted to participants’ body weight, which resulted in higher doses for individuals with above-average body weight, reaching close to 600 mg of caffeine.

The selection of exercise is of non-trivial significance for comparison of the results. In our project, a barbell multi-joint bench press exercise was utilized, while, in earlier research, a single-joint isokinetic dynamometer was used [7]. Significantly greater ergogenic effects of caffeine compared to MIPS and the lack of significant differences in subjective assessments of well-being, arousal and exhaustion make it proper to dispute the efficacy of MIPS supplementation. Another premise for the rejection of MIPS supplementation is the at least tenfold lower price of caffeine, as shown in our market query.

However, the question, of higher training volume performed with inter-set rest periods optimal for hypertrophy and the same intensity leading to better training adaptations remains open. With small ergogenic effect size (d < 0.05), detection of significant differences in training adaptations would require, as we already mentioned, a considerably bigger group of participants. Identification of such effects could be facilitated by a combination of a couple of ergogenic methods in a training protocol intended to develop adaptation in muscle strength, power, endurance and hypertrophy. 

Amongst other methods of facilitating ergogenic effects reported in literature in the strength training context, we can count post-activation potentiation [87], hyperventilation [88], intra-training body cooling [89] and ischemic preconditioning [90]. However, it remains to be shown if simultaneous use of these methods and supplements can lead to the accumulation of their ergogenic benefits, and future research in this direction is much needed.

## 5. Conclusions

In the context of our study, the answer to the question about the superiority of MIPS over anhydrous caffeine supplementation turned out to be negative for all outcomes and led to the following conclusions:

- In a group of resistance-trained men, supplementation of caffeine may lead to greater ergogenic benefits than MIPS with no differences in psychological aspects. This conclusion is aggravated by the results of the market query and prices of supplements in both categories, since:
- In the Polish market, the dose of caffeine is, on average, tenfold cheaper in the form of a pill and as much as 50-fold cheaper in the form of powder than MIPS. Results of this research may suggest a shift in supplementation habits of those participating in resistance exercise. It is supported by both the ecologically valid evaluation of the MIPS in our study and higher caffeine efficacy, as well as economic calculation. Nevertheless, more research on varied populations and products with different ingredient profiles may be needed to draw final conclusions about MIPS efficacy.

## Figures and Tables

**Table 1:** Producer’s suggested doses and mean used in the study—body-weight-adjusted doses of specific MIPS ingredients.

Ingredient	Suggested Dose (mg)	Mean Dose (mg)	±	SD
Beta-alanine	3000	4986	±	539
L-citrulline malate	3000	4986	±	539
Arginine alpha-ketoglutarate	1200	1994	±	216
L-taurine	1200	1994	±	216
L-tyrosine	1000	1662	±	180
Caffeine	300	499	±	54

SD—standard deviation.

**Table 2:** Effects of caffeine and multi-ingredient pre-workout supplement (MIPS) ingestion on bench press repetition volume.

Variable	Post-Caffeine ± SD			Post-MIPS ± SD			ES
Set 1 repetition volume	11.92	±	0.86	11.69	±	1.44	0.19
Set 2 repetition volume	8.15	±	1.46	8.15	±	1.34	-
Set 3 repetition volume	6.31	±	1.11 *	5.92	±	0.95 *	0.37
Set 4 repetition volume	5.31	±	1.03	5.15	±	1.07	0.15
Set 5 repetition volume	5.15	±	1.41	4.92	±	0.86	0.23
Total repetition volume	36.85	±	4.36 *	35.85	±	4.36 *	0.20

SD—standard deviation; ES—effect size; * *p* < 0.05.

**Table 3:** Effects of caffeine and multi-ingredient pre-workout supplement (MIPS) ingestion on pre- and post-training feeling scale (FS), felt arousal scale (FAS) and session rating of perceived exertion (sRPE).

Variable	Post-Caffeine ± SD			Post-MIPS ± SD			ES
Pre-training FS	3.88	±	0.92	3.54	±	1.31	0.31
Pre-training FAS	3.85	±	1.14	4.12	±	1.06	−0.24
Pre-training sRPE	2.58	±	1.61	2.88	±	1.31	−0.21
Post-training FS	2.85	±	2.17	3.54	±	1.35	−0.39
Post-training FAS	3.85	±	1.09	4.08	±	1.59	−0.17
Post-training sRPE	6.62	±	1.70	6.04	±	1.90	0.32

SD—standard deviation; ES—effect size.

**Table 4:** Supplement portion size, caffeine content and price per portion comparison.

Supplement	Mean Suggested Portion Size (g) ± SD	Mean Caffeine Content (mg) ± SD	Mean Price per Portion (PLN) ± SD
Local MIPS	13.2 ± 6.54	187.45 ± 90.3	2.52 ± 1.41
Imported MIPS	14.46 ± 7.95	202.91 ± 109.06	3.63 ± 1.78 *
PC caffeine	0.2	200	0.25 ± 0.07 **
Powder caffeine	0.2	200	0.05 ± 0.03 **

SD—standard deviation; PC—pill; MIPS—multi-ingredient pre-workout supplement; PLN—ISO 4217 code for Polish currency; * *p* < 0.05 compared to local MIPS; ** *p* < 0.001 compared to local MIPS.

## Data Availability

The data presented in this study are available on request from the corresponding author.
